# Modern Methods of Treatment of Compound Fracture

**Published:** 1894-02-10

**Authors:** 


### MODERN METHODS OF TREATMENT OF COMPOUND FRACTURES.

The practice of antiseptic surgery has revolutionised every department of surgery, but in none has this revolution been more striking and beneficial than in the treatment of compound fractures. To those who have only witnessed the results of to-day, with a percentage mortality of units only, it is almost incredible that thirty years ago the mortality attending the same class of injuries was from 50 to 80 per cent. Yet such is the fact, and a large number of the lives that were then saved were crippled by the loss of a limb, while to-day primary amputation for compound fracture is rarely needed. The saving of limbs is quite as striking as the saving of life. Not many years ago primary amputation for compound fracture of the leg and thigh was one of the commonest major operations —to-day it is one of the rarest. How rare it is, or at any rate should be, may be gathered from a lecture of Mr. Pearce Gould's, recently published in the *Clinical Journal*, in which it is laid down as a surgical maxim that "there is no condition in a compound fracture which demands amputation which, if existing in a simple fracture, would not also demand primary amputation." And it is shown that these conditions are only two—(1) such interference with the circulation that the part below cannot be kept alive, and (2) such injury to the soft parts that recovery with restoration of function is impossible. Mere comminution of a fracture, or its extension into a joint, or injury of one of two main arteries, are not sufficient grounds for amputation. This, then, is the twofold primary result of the antiseptic treatment—the preservation of life and the saving of limb, by the abolition of all the local and systemic evil results of septic infection. Having gained this much, there is one thing more to secure—the restoration of a perfect limb. This involves the perfect repair (1) of the bone, and (2) of the soft parts. The difficulty in securing perfect repair of the bone arises from the comminution and the displacement—over-riding or angular—of the fragments. Comminuted fragments should not be removed, but the greatest care should be taken to replace them with the utmost accuracy; they are often found widely displaced, turned round on their own axis, partially impacted, or perforating the adjacent muscles. All this must be corrected, and they must be replaced exactly, even if it take as much time and care as the piecing together of a puzzle. This, however, is only half the difficulty; there still remains the task of holding the fragments in apposition. And this may tax the surgeon's ingenuity to the full. In the lecture above referred to, Mr. Gould lays stress on the fact that one condition essential to success is perfect restoration of the fragments. A fracture that is not "reduced" cannot be "set." If the ends of the broken bone are not brought accurately into apposition, they cannot be held immovable for union to take place. But there are many cases of oblique fracture where, after exact fitting together of the bones, the weight of the limb and the pull of the muscles, quickly produces over-riding or angular displacement. How is this to be got over? One plan that has been largely practised is to wire together the fragments, or to secure them by metal or ivory nails. In certain cases one or other of these plans will succeed admirably. The wire suture has been used with great success in holding in apposition the fragments of a broken patella and of a broken lower jaw; it is sometimes successful in the humerus and femur. But there are two or three objections to it. One is the difficulty of passing the suture even when the fragments are properly drilled; another is that the suture often prevents displacement in one axis only, and leaves the fragments mobile in another; a third objection arises from the injurious effect of the firm pressure of the wire upon a linear portion of the bone, which may induce necrosis. The metal and ivory nails passed after drilling the fragments are useful, particularly if they can be passed in more than one axis, so as to prevent movement in all directions. More recently three other plans have been introduced. One of these is the intraosseous splint of ivory or bone, which we owe to German surgeons—Heine, Langenbeck, and Bircher. This is a kind of plug of bone or ivory of a suitable size, which is first thrust into the medullary cavity of one fragment, and then the other fragment is impaled on the other end of the plug in the same way. It acts as a central splint. It is of no use in cases of comminution, and by its presence it interferes to some extent with the natural process of healing of the fracture. Another plan comes to us from Dr. Senn, of Chicago: it is the use of a ring or ferrule of bone to hold the fragments together. These ferrules are prepared from the bones of oxen of approximately the same shape and size as those of men. A suitable one is chosen and slipped over one end of the broken bone; the two ends are then brought into exact apposition, and the ferrule is slipped down so as to embrace and fix both ends. Dr. Senn reports three cases in which he used these ferrules with good effect. We think that in many cases they will be very useful; where there is much comminution they will hardly meet the need, and the difficulty of preparing them and of having them of a proper size and shape may interfere with their extensive use. The third plan is British, and was incidentally mentioned by Mr. Watson Cheyne at a recent meeting of the Clinical Society. It consists of a plate of perforated aluminium, which is laid over the fragments and fixed to them by metal nails; the plate is buried in the wound, and remains permanently. This method also is not well adapted for great comminution.

Only second in importance to the bone is the perfect restoration of the soft parts. These should be replaced accurately and fixed in place by suitable sutures.

To sum up, the modern treatment of compound fractures is directed to four points: (1) To render and to keep the parts aseptic; (2) to preserve all viable limbs; (3) to restore the bone perfect in length and contour; (4) to secure primary and exact healing of all wounded soft parts.

Now it is evident that this constitutes an important surgical operation, and it should be looked upon in this light. It requires all the skill and patience of the surgeon; it should be undertaken deliberately and with full preparation of all details. An anæsthetic should be administered; if necessary, the wound should be enlarged, or a fresh wound may be made; every effort should be made to secure a perfect result. Modern surgery is a fine art, and this is shown in no case more perfectly than in the successful treatment of compound fractures.

